# Years of life lost to COVID-19 in 20 countries

**DOI:** 10.7189/jogh.12.05007

**Published:** 2022-03-28

**Authors:** Greg Williams, Angela Spencer, Tracey Farragher, Matthew Gittins, Arpana Verma

**Affiliations:** Division of Population Health, Health Services Research and Primary Care, The University of Manchester, Manchester, UK

## Abstract

**Background:**

Although the highest burden of COVID-19 mortality is in older age groups, there is considerable burden of premature mortality, including within older age groups who have died as a result of the novel disease. The aim of this work was to calculate years of life lost (YLL) to COVID-19 for countries with age and sex specific COVID-19 death data available and investigate the burden of premature mortality amongst the included countries. A secondary aim was to investigate the underestimation of YLL when using country specific life expectancies instead of global life expectancies.

**Methods:**

This study calculates YLL to COVID-19 for 20 countries to investigate the burden of premature mortality and underestimation of YLL when using country specific life expectancies compared to global. Population statistics and cumulative COVID-19 death data were extracted from the National Institute for Demographic Studies’ Demography of COVID-19 Deaths database. Overall YLL, YLL per 1000, cumulative YLL with age, and peak deaths per 1000 were calculated.

**Results:**

USA has the highest overall YLL with 10 289 624 compared to Norway with the lowest YLL of 10 771. When taking into account population size, South Korea has the lowest YLL at 0.55 per 1000 people, with Moldova having the highest at 49.63 per 1000 people. In terms of COVID-19 deaths per 1000 people, South Korea again has the lowest (0.04), but England & Wales have the highest (2.39). The USA, Ukraine, Moldova and Romania have a larger burden of YLL in younger ages. England and Wales had the highest loss to a population category, with 5.78% of those aged 90+ dying of COVID-19. When using local life expectancy instead of global estimates, the burden of YLL was underestimated by as much as 47.9%.

**Conclusions:**

This study highlights that although the higher burden of YLL is with older age groups, some countries have a high burden of YLL in younger age groups that should not be ignored. It also demonstrates that life should be valued across all age groups and geographies, and when making decisions locally, there is value in decision makers comparing local lives to globally optimal values.

The COVID-19 pandemic was declared a Public Health Emergency of International Concern (PHEIC) by the World Health Organization (WHO) on January 30, 2020 before being characterised as a pandemic on March 11, 2020 [1,2]. Since the first COVID-19 death, the disease has claimed over 4.25 million lives globally [3]. This is a truly global health issue, which crosses borders in terms of the disease as well as the solutions required [4].

The emergence of a novel disease was not wholly unexpected, with WHO adding a “Disease X” to their ‘List of Blueprint priority diseases’ in 2018, which represented an unknown disease likely to cause an international epidemic [5]. Although some nations developed plans based on the threat of Disease X, the COVID-19 pandemic demonstrates there are lessons to be learned from countries’ implementation of plans and the varying ways and timeframes of responses [6,7], likely contributing to the differing health outcomes [8-11].

Governments around the world provided measures such as physical distancing, with guidance for extra caution around those who are at highest risk from COVID-19, including older adults and other subgroups such as those with chronic diseases [12,13]. It is widely acknowledged that the risk of mortality from COVID-19 increases with age, with the highest death rates amongst older age groups [14,15]. As a novel disease, it is reasonable to suggest that deaths from COVID-19 occur earlier than would otherwise have happened. Most premature mortality measures use an upper endpoint for defining when a death is premature, such as 70 or 75 years of age [16-18]. However, these measures risk diminishing the impact of COVID-19 mortality amongst older populations and a measure of premature mortality without an upper endpoint should be considered.

Although the majority of deaths due to COVID-19 have been in older aged people worldwide, there has been a large number of deaths in younger age groups, so it is appropriate to investigate the years of life lost (YLL) in populations [19]. YLL measures premature mortality by calculating the amount of time lost between someone’s death and the potential maximum life expectancy expected to have been observed had they not died at that age [20]. The measurement also implies there is an amount of life lost at all age groups, avoiding arbitrary cut-offs whilst still providing greater weight to deaths occurring at a younger age [20]. The Global Burden of Disease, Injuries and Risk Factors study (GBD), which is the largest global study looking at health loss due to diseases, injuries and risk factors [21], estimate YLL in order to help support public health planning and policy [20].

YLL calculations take into account the remaining life expectancy that someone would have been expected to achieve had they not passed away at that age [21]. The GBD provides estimates of life expectancy for all ages up to 109 years old in 401 countries and regions of the world. As life expectancy estimates vary widely across the world (GBD’s <1 year life expectancy ranges from 54.9 years in the Central African Republic to 88 years in the Nagano region of Japan), it is important to understand the difference in YLL estimates where the country specific life expectancy is used compared to a global highest to understand how the burden of premature mortality may be downplayed, and the lower life expectancy may be unfairly ‘accepted’ based on geography. This approach towards understanding COVID-19 mortality also provides an alternative narrative to recognise how different countries have coped with the reality of the pandemic compared with how they were expected to cope through measures such as the Global Health Security Index, which found in 2019 that the United States and the United Kingdom were the two most prepared countries for an epidemic or pandemic [22].

The aim of this work was to calculate YLL to COVID-19 for countries with age specific COVID-19 death data available and investigate the burden of premature mortality amongst the included countries. A secondary aim was to investigate the underestimation of YLL when using country specific life expectancies instead of global life expectancies.

## METHODS

Population statistics and cumulative COVID-19 death data were extracted from the National Institute for Demographic Studies (INED)’s Demography of COVID-19 Deaths database for 20 countries on 4 August 2021 [23]. All countries with data available for age specific COVID-19 deaths were included in the study. Female and male life tables were extracted from the Global Burden of Disease Study 2019 Life Tables 1950-2019 [24] for all countries in the world to identify the highest life expectancies at different ages. The GBD reference life table was not used as all age groups are not included, and this study aims to investigate YLL against the global and included country maximum life expectancies. The age of deaths were grouped differently depending on which country the data came from (eg, Belgium’s data had six age categories which largely cover 20 years each at younger ages and 10 years from 65, whereas England & Wales covered 5 years per category, up to 90+). Therefore, the median age point for each category was used for the reference remaining life expectancy with the oldest age point chosen as a continuation of the preceding pattern of age points (eg, 75, 85, 95). A remaining life expectancy (RLE) was assigned to each age point, including the sex specific RLE for that country, the highest RLE for the countries included in the study, and the highest RLE in the world.

YLL was calculated using the formula YLL = N × L, where N is the number of reported COVID-19 deaths within the age and sex group and L is the life expectancy at the age of death (RLE) [25]. YLL was calculated for each age category and sex, with the three different L estimations: 1) RLE for the specific country/age/sex, 2) the RLE for the highest country/age/sex in the study and 3) the RLE for the highest country/age/sex in the world. Comparisons were made between the reference RLEs to understand the underestimation of YLL when using local (country) RLE estimates compared to global RLE estimates. Using the highest remaining life expectancy globally for calculating the YLL is in keeping with GBD methodology, as it allows for greater comparison between the included countries and is justified as an egalitarian approach by not valuing someone’s contribution to the global burden of disease as higher in one country than another [26,27].

All YLLs were calculated sex specific and against the highest RLE for that estimation. No age weighting or discounting was applied so as not to value one year of life in the future as less valuable than another, as adopted by WHO and the GBD following expert consultation [28,29]. Overall YLLs were calculated for each country as well as each age category, using the age specific highest life expectancy for the country, countries in the study, and the world. Age and sex specific population figures were used to calculate YLL per 1000 for each category. COVID-19 deaths per 1000 were also calculated for each category.

In order to explore the burden of premature mortality due to COVID-19 in the included countries, rank comparisons were made between COVID-19 deaths per 1000 and the YLL per 1000. Cumulative deaths by increasing age category were also used to explore premature mortality, including accounting for age specific population size. The age categories with the maximum YLL, peak deaths from COVID-19 and peak deaths as a proportion of the age-specific population were also calculated and reported in order to provide more context to the burden of COVID-19 mortality in the included countries.

Where countries had breakdown of deaths by age but not sex, only overall YLL for that age category was calculated. An additional “unknown” category was included for countries who had an additional group whose sex was not reported, these figures were only included in the overall YLL figures.

All calculations were done in Microsoft Excel 2016 (Microsoft Inc, Seattle WA, USA) [30] and results were reported in accordance with the GATHER statement [31].

## RESULTS

Of the 20 countries included in this study, COVID-19 deaths with an age and sex breakdown were available for all except three (Romania, South Korea and Sweden), which only had the age breakdown. Table 1 shows the YLLs and YLLs per 1000 population for each country against each reference RLE, the total COVID-19 deaths, deaths per 1000, population size, COVID-19 deaths and YLL per 1000 rank between the countries, the rank difference, and the % difference in YLL per 1000 between using the highest life expectancy of the individual country and the global highest life expectancy.

**Table 1:** Overall years of life lost (YLLs) to COVID-19, YLL per 1000 persons, COVID-19 deaths, rankings and difference in YLL estimates for the 20 included countries*

	Years of Life Lost (YLL) to COVID-19 Local (highest)	YLL to COVID-19 included countries (highest)	YLL to COVID-19 world (highest)	YLL to COVID-19 Local (highest) per 1000	YLL to COVID-19 included countries (highest) per 1000	YLL to COVID-19 world (highest) per 1000	Total COVID-19 deaths	Total population	COVID-19 Deaths per 1000	COVID-19 Deaths rank (per 1000)	YLL to COVID-19 rank (per 1000) at world highest LE	Rank difference	% difference in Local to world LE estimation
**Austria**	107 449	120 312	132 782	12.13	13.58	14.99	10 534	8 858 775	1.19	7	8	-1	19.08
**Belgium**	249 149	275 668	305 242	21.68	23.99	26.56	25 231	11 492 641	2.20	19	15	4	18.38
**Canada**	287 299	298 592	332 324	7.56	7.86	8.74	26 071	38 005 238	0.69	4	4	0	13.55
**Denmark**	24 494	28 026	30 967	4.21	4.81	5.32	2540	5 822 763	0.44	3	3	0	20.90
**England & Wales**	1 567 413	1 780 346	1 951 913	26.51	30.12	33.02	141 407	59 115 809	2.39	20	18	2	19.70
**France**	988 605	990 667	1 089 552	14.74	14.77	16.25	85 281	67 063 703	1.27	10	9	1	9.26
**Germany**	875180	1 016 288	1 124 049	10.54	12.24	13.54	91 565	83 019 213	1.10	6	6	0	22.14
**Italy**	1 406 486	1 488 385	1 638 651	23.30	24.66	27.15	127 044	60 359 546	2.10	17	16	1	14.17
**Moldova**	96 221	123 501	131 044	36.44	46.77	49.63	5772	2 640 438	2.19	18	20	-2	26.57
**Netherlands**	171 062	197 903	218 954	9.90	11.45	12.67	17 804	17 282 163	1.03	5	5	0	21.87
**Norway**	8980	9854	10 771	1.67	1.84	2.01	796	5 367 580	0.15	2	2	0	16.63
**Portugal**	182 828	206 786	229 833	17.79	20.12	22.36	17 320	10 276 617	1.69	12	11	1	20.45
**Romania**	491 388	635 896	681 225	25.42	32.90	35.24	34 275	19 328 838	1.77	14	19	-5	27.87
**Scotland**	109 391	130 597	142 929	20.02	23.90	26.16	10 220	5 463 300	1.87	16	14	2	23.46
**South Korea**	24 192	25 744	28 340	0.47	0.50	0.55	1969	51 847 509	0.04	1	1	0	14.64
**Spain**	958 864	994 971	1 101 984	20.36	21.12	23.40	80 748	47 100 395	1.71	13	12	1	12.99
**Sweden**	139 308	155 190	171 928	13.49	15.03	16.65	14 656	10 327 553	1.42	11	10	1	18.97
**Switzerland**	108 276	113 784	127 543	12.62	13.26	14.86	10 341	8 582 905	1.20	9	7	2	15.11
**Ukraine**	736 749	995 348	1 061 197	17.65	23.85	25.43	50 058	41 732 779	1.20	8	13	-5	30.57
**USA**	8 496 660	9 540 713	10 289 624	25.97	29.16	31.45	603 516	327 167 434	1.84	15	17	-2	17.42

*Calculated with population and life expectancy data from the Global Burden of Disease Study 2019 [24] and COVID-19 mortality data from INED [23].

### Overall YLL

The United States has the highest overall YLL with 10 289 624 compared to Norway with the lowest YLL of 10 771. When taking into account population size, South Korea has the lowest YLL at 0.55 per 1000 people, with Moldova having the highest at 49.63 per 1000 people. In terms of COVID-19 deaths per 1000 people, South Korea again has the lowest (0.04), but England & Wales have the highest (2.39).

When ranking the deaths per 1000 against the YLL per 1000, the lowest six countries ranked the same. Ranking was improved by one place for five countries when considering YLL per 1000. One country (Belgium) ranked higher by 4 places (19^th^ to 15^th^), and three countries 2 places (England & Wales, Scotland and Switzerland). Romania and Ukraine (5 places), and the United States and Moldova (2 places) all ranked lower of the included countries when looking at YLL per 1000 compared to deaths per 1000, indicating a higher burden of premature mortality from COVID-19 in these countries.

### YLL calculated using different RLEs

When comparing the burden of YLL using local RLE as opposed to global (optimal) RLE per age category, the YLL per 1000 of the population was underestimated by as much as 30.57% in the Ukraine, with France having the lowest underestimation of 9.26%. However, this figure still uses the highest local RLE by sex for the age categories, which is female for all countries and age categories in the study except for Germany in age group 90+. If the YLL is calculated using the male population and local male RLE only, the underestimation is as much as 47.9% for the Ukraine (14.5 per 1000 compared to 27.9 per 1000) and 31% calculated using the female population (23.3 per 1000 compared to 16.1 per 1000). This sex specific underestimation pattern is similar for other countries, for example, YLL for France’s male population is underestimated by 24.6% (20.8 per 1000 compared to 15.7 per 1000) but only 10% for their female population (11.8 per 1000 compared to 10.6 per 1000).

### Cumulative YLL

**Figure 1:**
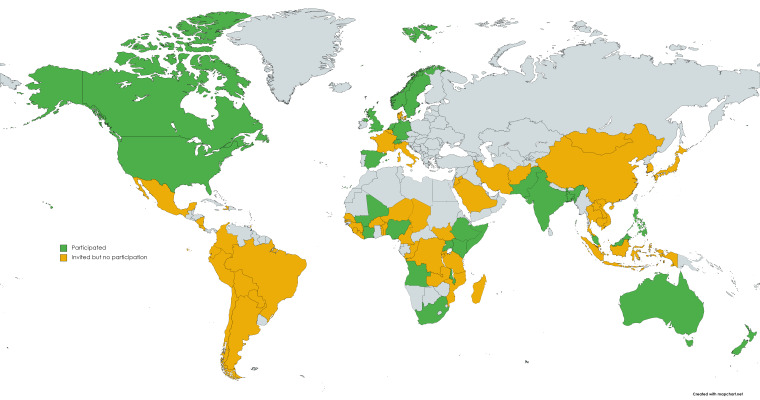
Cumulative years of life lost (YLLs) to COVID-19 for 20 countries. Calculated with population and life expectancy data from the Global Burden of Disease Study 2019 (24) and COVID-19 mortality data from INED [23].

Figure 1 shows the cumulative YLL for the included countries. The United States has a noticeably higher YLL than the other countries, largely owing to its larger population size with the other countries in the study having a population size of between 0.8% (Moldova) and 25.4% (Germany) of the US. However, the USA has a higher burden of YLL at younger ages than most other included countries. In cumulative YLL terms, the USA has a higher burden of YLL by ages 15-24 than Denmark, Norway and South Korea have in their whole individual populations. By age category 25-34, this burden is larger than in Austria, Belgium, Canada, Denmark, Moldova, the Netherlands, Norway, Portugal, Scotland, South Korea, Sweden and Switzerland.

**Figure 2:**
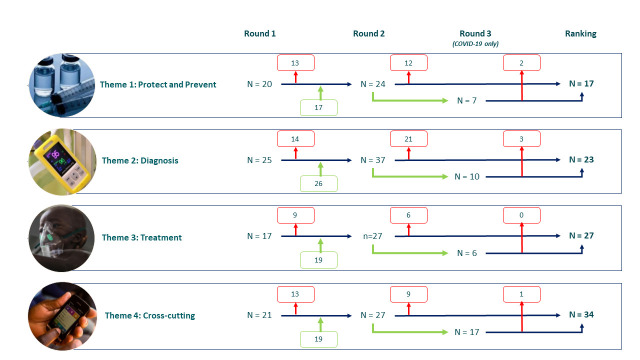
Cumulative years of life lost (YLLs) to COVID-19 per 1000 people against cumulative population up to age 50 years. Calculated with population and life expectancy data from the Global Burden of Disease Study 2019 [24] and COVID-19 mortality data from INED [23].

**Figure 3:**
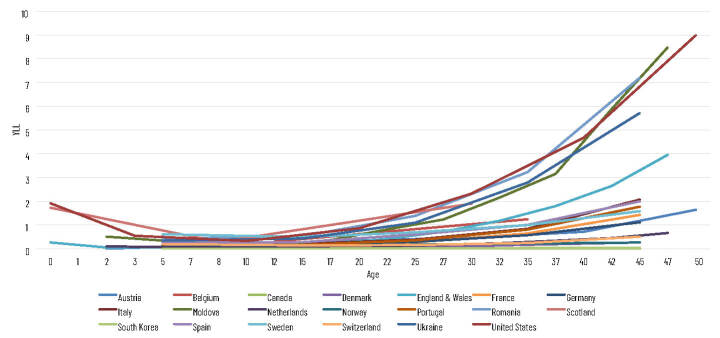
Cumulative years of life lost (YLLs) to COVID-19 per 1000 for all ages. Calculated with population and life expectancy data from the Global Burden of Disease Study 2019 [24] and COVID-19 mortality data from INED [23].

When taking the cumulative population size into account (Figure 2 and Figure 3) the US, Ukraine, Romania and Moldova are considerably higher than the other countries by median age point 50 with the Ukraine at 5.72 YLL per 1000 (age group 40-49), Moldova 8.49 YLL per 1000 (45-49), Romania 7.20 YLL per 1000 (40-49) and the USA 9.00 YLL per 1000 (45-54). England & Wales and Italy are the only other countries larger than 2 YLL per 1000 with 3.96 YLL per 1000 (45-49) and 2.07 YLL per 1000 (40-49) respectively. As per Table 1, Figure 3 shows all age groups, with Moldova having the highest YLL per 1000 (49.63), and South Korea (0.55), Norway (2.01), Denmark (5.32) and Canada (8.74) having the lowest YLL per 1000 people.

This trend of the US, Moldova, Romania and Ukraine having a higher burden of YLL in younger age groups is echoed in overall terms by Table 2 which shows the age groups with the maximum burden of YLL per country. The USA (2 744 468 YLL) experiences its highest YLL in 65-74-year olds, Moldova (27,626.9) experiencing it in the 65-69-year old category, Romania (214,116.8) in 60-69 and Ukraine (361,432) in 60-69. However, when taking into account population size the burden is in younger age categories for Moldova, Romania and Ukraine compared with other countries. Table 2 also shows the age range with the peak deaths from COVID-19, with Moldova experiencing their peak (1203 deaths) at 65-69 years of age. When peak deaths are reported as a proportion of the population for that age group, England & Wales report the highest proportion of deaths per age group overall with 5.78% of 90+ year olds dying as a result of COVID-19. South Korea’s peak age category shows the lowest proportion of deaths (in the highest category for the country) with 0.06% of 80+ year olds dying from COVID-19. All countries except for Moldova and Ukraine experienced their peak deaths as a proportion of the population in the oldest age group reported.

**Table 2:** Maximum years of life lost (YLLs) and peak deaths, including proportion of the population from COVID-19 for the 20 included countries*

Country	Max YLL	Max YLL age range	Max YLL per 1000	Max YLL per 1000 age range	Peak deaths from COVID-19	Peak deaths from COVID-19 age range	Peak deaths as proportion of population	Peak deaths as proportion of population age range
Austria	44 687.67	75-84	129.33	85+	4584	85+	2.02	85+
Belgium	92 545.92	75-84	239.91	85+	12 613	85+	3.76	85+
Canada	152 862.10	80+	91.88	80+	16 695	80+	1.00	80+
Denmark	11 063.22	70-79	51.49	90+	996	80-89	1.26	90+
England & Wales	283 129.00	80-84	312.65	90+	30 587	90+	5.78	90+
France	314 690.70	70-79	92.44	80-89	32755	80-89	2.08	90+
Germany	371 932.30	80-89	100.95	90+	40 621	80-89	2.46	90+
Italy	522 382.70	70-79	132.02	80-89	51 267	80-89	3.18	90+
Moldova	27 626.90	65-69	219.21	70-74	1203	65-69	1.26	80-84
Netherlands	40 858.33	75-79	141.49	90-94	4009	85-89	3.87	95+
Norway	2981.69	70-79	17.75	90+	265	80-89	0.43	90+
Portugal	103 785.01	80+	156.90	80+	11 335	80+	1.71	80+
Romania	214 116.80	60-69	120.55	70-79	11 328	70-79	1.37	80+
Scotland	41 317.37	75-84	204.35	85+	4074	85+	3.21	85+
South Korea	9943.59	80+	5.24	80+	1086	80+	0.06	80+
Spain	464 803.20	80+	162.46	80+	50 764	80+	1.77	80+
Sweden	54 634.80	80-89	156.35	90+	5967	80-89	3.82	90+
Switzerland	66 812.49	80+	150.10	80+	7297	80+	1.64	80+
Ukraine	361 435.00	60-69	87.8	70-79	15 559	70-79	0.60	80-89
USA	2 744 468.00	65-74	173.94	85+	178 572	85+	2.73	85+

*Calculated with population and life expectancy data from the Global Burden of Disease Study 2019 [24] and COVID-19 mortality data from INED [23].

## DISCUSSION

This study demonstrated that of the included countries, the USA has the highest overall burden of YLL to COVID-19. This is due to the population size, however, when adjusted for size the USA still compares unfavourably with the 17^th^ largest YLL per 1000 out of the 20 included countries, with only England & Wales, Romania and Moldova ranking lower. The study also showed that the US, Ukraine, Moldova and Romania have a larger burden of YLL in younger age groups (<50 years) than the other included countries. Where peak COVID-19 deaths were looked at as a proportion of the population for the particular age range, England and Wales had the highest loss to a population category with 5.78% of the over 90+ population losing their life to COVID-19, nearly 2% higher than the next largest population loss of 3.87% in the Netherlands 95+ age group.

This study also showed the differences when calculating YLL using country specific RLEs compared to using the global maximum for each age category. The underestimation of YLL using country specific RLE was inevitable due to none of the included countries having the highest life expectancy in the world. However, the size of the underestimation is important when YLLs are utilised in decision making processes. National and subnational data can be useful for informing health policy at the level policy making is devolved to [32] and multi-country studies designed for comparability like the GBD may not have the data available that national teams have, so their approaches can differ, resulting in different estimations. An example of this is the Scottish Global Burden of Disease Study, which used a counting approach compared to the GBD’s modelling approach [33,34]. Although a local data-driven approach can help to inform local policy, it can be difficult to make comparisons with other countries where indicators and methodologies differ. By using country or subnational specific data in calculating YLLs, decision makers could be inadvertently justifying the inequalities experienced locally and valuing their own population’s life as less than that of another part of the world [35]. This may diminish the scale of the problem to decision makers, potentially impacting on the allocation of resources.

Our study found that when taking into account population size, the United States and Moldova had the higher burden of YLL in younger age groups. The United States has suffered from higher levels of premature mortality than other comparable High Income Countries prior to the COVID-19 pandemic, however it has lower death rates in the 80+ age categories than most comparable countries in recent decades [36]. Studies have shown there to be differences in mortality amongst different ethnic groups due to COVID-19 [37,38], and a cross-sectional study looking at racial/ethnic disparities in COVID-19 mortality in the USA found there to be a higher burden of premature mortality in non-Hispanic Black and Hispanic populations compared with the non-Hispanic White population [39]. Further investigation into YLL for different ethnic groups to identify the burden of premature mortality in more detail would be useful to help target preventative approaches and initiatives.

Our study was limited to the 20 countries with available age-specific COVID-19 death data. Of the 20 countries involved, 17 of the countries are classified as High Income Countries according to the World Bank, with Moldova and Romania classified as Upper-Middle Income Countries, and Ukraine classified as a Lower-Middle Income Country [40]. Of the countries in the study, with the exception of the United States, we found that the included High Income Countries had a higher portion of YLL due to COVID-19 in the older populations in overall terms, compared with the Lower-Middle Income Countries in the study who had a higher burden in younger age groups. This pattern was also observed in a similar study looking at YLL due to COVID-19 [41].

We also found that England & Wales had the highest peak deaths as a proportion of the population to COVID-19, losing 5.78% of its 90+ population to COVID-19, compared to the lowest with 0.06% of the 80+ population in South Korea. The large increase in deaths in care homes in England & Wales will contribute to this figure, with an estimated 79% increase in the first three months of the pandemic [42]. When compared with care home deaths in Australia, which has a similar health care system, demographics and care home settings, one study found that the fatality in the UK was 270-300 times worse, implying the difference in approach to lockdown could have been a major contributory factor to this high death rate [11].

This study has only looked at mortality related to COVID-19 but many of those infected may suffer from long-term physical and mental health outcomes as a result of the disease, including immunological consequences making them more susceptible to other diseases [43]. Therefore, it would be appropriate to not only consider the YLL when looking at the impact of the pandemic, but also to consider Disability-Adjusted Life Years (DALY), which considers the difference between the current state of health and the most ideal state of health by adding Years Lived with Disability (YLD) to YLL [25].

### Limitations

Due to differences in testing and reporting of COVID-19 between countries, the use of excess all-cause mortality may provide a more accurate representation of the true COVID-19 mortality and therefore YLL [44]. This would also include deaths of those who have not died directly of COVID-19 but were it not for the pandemic, along with the subsequent measures to control it, they would still be alive (or have died at a later date and have a lower RLE). A recent study which included analysis of YLL due to COVID-19 against excess deaths for 81 countries found that the true burden of mortality due to COVID-19 is likely to be much higher, with countries on average underestimating the YLL due to COVID-19 by a factor of 3, but by more than 12 in some countries [41]. However, studies acknowledge that the mortality figures include people who were vulnerable with long term conditions and multi-morbidities, whose life expectancy may have been lower anyway [45]. This implies that the YLL calculations used in our study could be an overestimation, however, were they to still pass away due to another ailment, then YLL calculations would still include their premature mortality against an optimal life expectancy at age of death, so our study includes this difference in YLL attributable to COVID-19.

The countries involved in the study report the age of deaths in groups as opposed to year specific and so the YLL calculations will not be as accurate as they could be were year specific data available. This accuracy will also vary between countries with some reporting 5-year age categories all the way through and other countries having categories as wide as 20 years, impacting the accuracy through the use of a median age. This study was also limited to the countries where data were available through INED’s website [23] and future studies would benefit from including more countries if data were available.

Additional challenges with the data comparison between countries includes the variation in methodologies for the collection of data of confirmed COVID-19 deaths. Four of the included countries changed their definition of death due to COVID-19 (Austria, Belgium, Denmark, England & Wales) since the first reported death. All countries except Italy and the USA used laboratory confirmation for the identification of COVID-19 deaths. In Italy information regarding COVID-19 laboratory confirmation is restricted and a positive PCR diagnosed by regional laboratories was used instead. The USA only used confirmation as “identified as cause of death”. Although coverage of the death is “all places” (eg, hospitals, care homes, other places) for the majority of countries, Moldova and Ukraine only include data from hospitals. In addition, the coverage of Austria, Canada and Italy was classed as “incomplete”. This is demonstrated as this data set reports the lowest age group with a death in Canada as 50-59, whereas the Public Health Agency of Canada’s Public Health Infobase reports 565 deaths in younger age categories as of 3^rd^ August 2021 [46]. However, with this being an ongoing pandemic it is important to use what data are available to make international comparisons that can be of value for future studies.

## CONCLUSION

Although older age has been a factor associated with higher mortality that has guided the public messaging and response to the pandemic, this study shows that there is a burden of YLL due to premature mortality in younger age groups, which should not be ignored in resource allocation, decision making and future studies looking at COVID-19 mortality. When calculating and interpreting YLL in future studies it is important to recognise the impact that different reference life expectancies will have on the data, particularly when understanding the burden of mortality in a population for decision making to ensure that inequalities due to geography are known. This study also demonstrates that we should not diminish the value of life lost in older age categories, and highlights that all mortality associated with COVID-19 is a loss, regardless of age.
